# Formulation and Characterization of Alginate-Based Membranes for the Potential Transdermal Delivery of Methotrexate

**DOI:** 10.3390/polym13010161

**Published:** 2021-01-04

**Authors:** Dorothea Bajas, Gabriela Vlase, Mădălina Mateescu, Oana Alexandra Grad, Mădălin Bunoiu, Titus Vlase, Claudiu Avram

**Affiliations:** 1 Research Centre for Thermal Analysis in Environmental Problems, West University of Timisoara, Pestalozzi Street 16, 300115 Timişoara, Romania; dorothea.bajas@gmail.com (D.B.); gabriela.vlase@e-uvt.ro (G.V.); madalina.mateescu@e-uvt.ro (M.M.); 2 Research Institute for Renewable Energy, Politehnica University of Timişoara, Piata Victoriei No. 2, 300006 Timişoara, Romania; oana.grad@upt.ro; 3 Faculty of Physics, West University of Timisoara, B-dul V. Parvan No. 4, 300223 Timişoara, Romania; 4 Physical Therapy and Special Motricity Department, West University of Timisoara, B-dul V. Parvan No. 4, 300223 Timişoara, Romania; claudiu.avram@e-uvt.ro

**Keywords:** drug delivery, methotrexate, membranes, TGA, FTIR analysis, UV-Vis study, SEM investigation

## Abstract

The aim of this study is to obtain and characterize of alginate-based membranes, as well as to choose the most suitable membrane type for the transdermal release of methotrexate. The paper presents the synthesis of four types of membranes based on alginate to which are added other copolymers (Carbopol, Tween, and Polyvinylpyrrolidone) as well as other components with different roles. Membranes and binary mixtures made between the components used in membrane synthesis and methotrexate are analyzed by thermogravimetric techniques, FTIR and UV spectroscopic techniques as well as SEM. The analyses aim to establish the type of membrane most indicated in the use of the controlled release of methotrexate, namely those membranes in which there are no interactions that could inactivate the active substance. Following these studies, it was concluded that membranes obtained from alginate/alginate and Tw can be used for methotrexate release. The membrane obtained from alginate and carbopol was excluded from the beginning because it is not homogeneous. Regarding the AGP-MTX membrane, it presents interactions with the active substance, carboxylate group interactions argued by TGA and FTIR studies, and interactions that occur in aqueous medium.

## 1. Introduction

The present study aims to evaluate the possibility of using different alginate-based matrix system for transdermal delivery of methotrexate (MTX). Low-dose methotrexate is an inexpensive, effective and widely used treatment as immunosuppressant drug for inflammatory conditions, including rheumatoid arthritis, psoriatic arthritis and juvenile idiopathic arthritis and as anticancer drug. TGA (thermogravimetric analysis), DSC (differential scanning calorimetry), and FT-IR (infrared spectroscopy with transformed Fourier) studies have been used to highlight the degree of incorporation of the active substance in the membrane and the possible interactions between the drug and the polymer. Transdermal drug delivery permits the use of a relatively potent drug with minimal risk of systemic toxicity and avoids the gastrointestinal degradation and hepatic first-pass metabolism [1].

Polymers are one of the major chemical compounds which find an important place in the field of engineering, medicine and pharmaceutical field these days and the major focus has been on the biodegradable ones, alginates in particular [2].

Alginates are polysaccharides found in the brown algae [3]. Alginates available for industrial use include sodium alginate, potassium alginate, mixed sodium-calcium alginate, salt of alginic acid and propylene glycol alginates. They are natural gums and offer advantages over synthetic polymers as they form hydrogels and they are non-toxic, biocompatible, biodegradable, less expensive and easily available. All these advantages make alginates a very useful material for biomedical applications, especially for the controlled delivery of drugs and other biologically active compounds.

MTX is used to control severe inflammatory arthritis such as rheumatoid arthritis psoriasis, juvenile rheumatoid arthritis, or to treat certain types of cancer [4].

Rheumatoid arthritis (RA) is the most common autoimmune inflammatory chronic arthritis in adults [5]. RA has a significant negative impact on the ability to perform daily activities, including work and household tasks, and health-related quality of life, and it increases mortality. It is characterized by progressive and irreversible damage of the synovial-lined joints, resulting in the loss of joint space, bone, and a decrease in joint function and deformity [5]. 

MTX belongs to a class of drugs known as antimetabolites. It is a type of disease-modifying anti-rheumatic drug (DMARD) frequently used in the treatment of inflammatory arthritis, although the exact mechanism of action is still unclear. Last available recommendations for the management of rheumatoid arthritis patients with synthetic disease-modifying antirheumatic drugs (csDMARD) released in 2016 by European League Against Rheumatism (EULAR) stated that: (1) MTX should be part of the first treatment strategy and (2) MTX should be the first csDMARD, either as monotherapy or in combination with other csDMARDs [6].This recommendations is based on MTX efficacy, safety, the possibility to individualize dose and method of administration as well as relatively low costs. MTX continues to be the anchor (‘first’) drug for patients with RA both as monotherapy as well as in combination with other drugs. Moreover, MTX appears to reduce comorbidities and mortality in RA [6,7]. In clinical trials MTX monotherapy has been associated with 25% response rates (which brings patients into the range of low disease activity) within six months [6]. 

The efficacy of MTX is related to its cytotoxic and anti-inflammatory effects when administered topical [8,9]. Several studies have compared transdermal MTX transport from different vehicles, but the data are quite controversial; this may be due to several factors such as influence of the components on the skin barrier properties, on the different species and types of skin and on different experimental procedures used for study [10]. Other studies show that methotrexate may be readily incorporated into oil-in-water emulsion. It remains stable up to a concentration of 0.5% and releases enough active substance to achieve relevant systemic bioavailability. There is no evidence of safety risks due to relevant systemic bioavailability after topical application on a limited area of the skin [11]. MTX effectively suppresses inflammation in arthritis and the systemic toxicity effects such as stomatitis, nausea, bone marrow depression, and liver toxicity are avoided [11]. The present study aims at several stages, namely: first of all, the synthesis of some membranes based on alginates and other copolymers that will have methotrexate incorporated as active substance. To ensure that the methotrexate does not suffer any interaction with the membrane components, the individual components used in membrane synthesis will be analyzed by TGA/DTG/DTA(HF) and FTIR techniques, followed by the study of binary mixtures made between the active substance methotrexate, obtained by evaporation of water from drug solutions used in the synthesis of membranes, and the individual components used in the synthesis of membranes.

In this paper, TGA/DTG/HF(heat flow) and FT-IR will be used to study Methotrexate -pure active substance (MTXSA), specifically Methotrexate obtained by the evaporation of injectable solutions (MTX) used in the synthesis of membranes, in order to verify whether the active substance obtained from the evaporation of the solution, namely the active substance used in the synthesis of membranes, contains the active principle in unaltered form. The study of the individual components used to obtain different types of membranes as well as MTX binary mixtures (from solution) with all components to see if there are any interactions. The study aims to identify possible interactions between components and validate the use of MTX-based injectable solutions as a source of MTX in membranes. The membranes obtained were also analyzed by TGA, FTIR, SEM (scanning electron microscopy), and UV-VIS (ultraviolet-visible) spectroscopy.

## 2. Materials and Methods

### 2.1. Chemicals and Reagents

**Figure 1:**
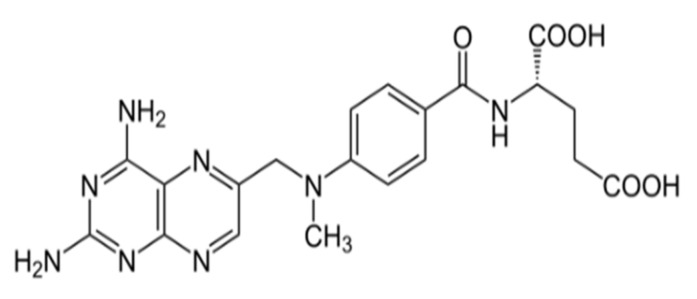
Structure of methotrexate.

Methotrexate MTX(SA): (2S)-2-[[4-[(2,4-diaminopteridin-6-yl)methyl-methylamino]benzoyl] amino] pentanedioic acid (Figure 1).

MTX (SA) was purchased from CALBIOCHEM (Cat: 454126, Lot: D00135520, MTX–EMD Chemicals, Inc. San Diego, CA, USA), Solution with MTX 10 mg/mL EBEVE Pharma Unterach Austria, Alginate from Sigma Aldrich P.N., (Saint Louis, MO, USA, W201502), Polyvinylpyrrolidone, M.W. 40000 powder (Calbiochem, Merck, Darmstadt, Germany, Lot: BCBV6638, CAS: 9003-39-8), Glicerin- Chimic Reactiv SRL, Tween^®^20 from Merck (Darmstadt, Germany, Lot: 17K034023, CAS: 9005-64-5); Carbopol^®^940 from ACROS ORGANICS (Geel, Belgium, Lot: A0386621, CAS: 9003-01-4).

### 2.2. Synthesis of Membranes

Alginate membrane was prepared by dissolving sodium alginate in water, while stirring 2h using a magnetic stirrer, at room temperature.

The topical membrane was prepared by dissolving PVP in mixture of acetone and isopropyl alcohol (40:60). Other co-polymers like Tween 20, Carbopol dissolved in different solvents in which the substances have maximum solubility were used. Glycerol was added as plasticizer.

Under gentle mechanical stirring, MTX solution was poured into the previously made polymer gel in a 2:1 ratio. The suspension was allowed to hydrate overnight and tomorrow day for gelling. Afterwards, the formed membranes were put at 50 °C for 1 h.

Obtaining binary mixtures: In order to highlight possible interactions between the active substance methotrexate in powder form, obtained from MTX injectable solutions, and the individual components used in the synthesis of membranes, binary mixtures were made in a mass ratio of 1:1. These mixtures were left for one week under dark conditions and 10 °C temperature to avoid degradation of the active substance in the two batches. The mixtures were analyzed by thermal analysis and FTIR spectroscopy comparing the results obtained in the case of the mixtures with the results obtained on the individual components.

### 2.3. Thermogravimetrical Analysis

TGA/DTA measurements were performed on a Perkin-Elmer (Waltham, MA, USA) DIAMOND thermobalance for obtaining simultaneously the TGA, DTG and HF curves. The thermal behavior was recorded in air (Linde Gaz Timişoara, 5.0) atmosphere with a flow rate of 100 mL∙min^−1^, temperature range was 25–500 °C with a heating rate of 10 °C min^−1^. Samples with mass between 5.0 and 15.0 mg were added to open aluminum crucibles.

### 2.4. FT-IR Spectroscopy

The FT-IR spectrums were recorded with a Perkin-Elmer (Waltham, MA, USA) 100 Spectrometer with U-ATR (universal total attenuated reflectance) technique in range of wave number 650–4000 cm^−1^. 

### 2.5. UV-Vis Spectrophotometry

The UV-Vis spectra were obtained using T90+ UV-Vis Spectrophotometer with double beam in photometric range: 190–900nm. All absorbance measurements were taken in a 10 mm UV/Vis spectroscopy cell at room temperature, using distilled water as a blank.

### 2.6. SEM Analysis

SEM analysis was performed with Quanta FEI (Hillsboro, OR, USA) 250 devices.

## 3. Results

### 3.1. Obtaining the Membranes

Following the general recipe presented above, the membranes without active substance and the membranes with active substance were obtained in order to compare the results obtained by the techniques listed above in the case of membranes with active substance and those without active substance, thus highlighting the presence of MTX in membranes. At the same time, it was desired to compare the membranes obtained on the basis of alginate, respectively, alginate and another copolymer tracking which membranes meet all requirements, namely elasticity, homogeneity, bioavailability of the active principle, and a lack of interactions between polymer components and active substance. Following the syntheses, it is found that the membranes containing Carbopol as a copolymer are not homogeneous and therefore, they will not be analyzed by physico-chemical methods (see Table 1).

### 3.2. Thermal Analysis 

**Figure 2:**
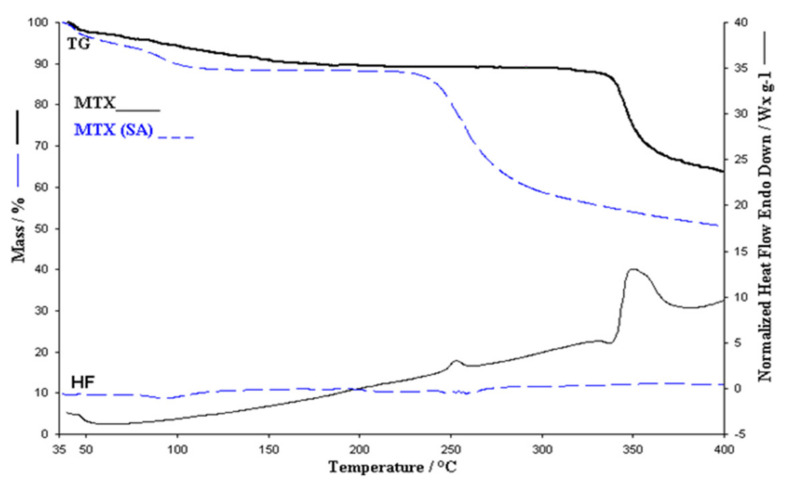
TG (Thermogravimetric) and Heat Flow (HF) curves of MTX and MTX(SA).

The thermal behavior of the sample MTX (SA) and MTX obtained by recrystallization from the injectable solution 40 mg mL^−1^ was studied in the air atmosphere by thermal analysis (TGA, DTG, HF), in order to highlight the changes that may occur during heating and also the thermal effects that accompany the decomposition processes (Figure 2). Thermal analysis is performed in the temperature range 35–400 °C in order to highlight the decomposition of the two forms of MTX, namely the active substance MTX (SA) and MTX, in order to reveal a possible different thermal behavior of the two forms as well as in order to validate the use of solutions in the synthesis of membranes. Thermal analysis of MTX (SA) showed a four-stage decomposition at a heating rate of β = 10 °C min^−1^ in air atmosphere. In the case of the active substance, the first two processes start at 35 °C and 70 °C, with mass loss of 4% and 8% respectively with maxima on the DTG curve at 38.5 °C and 91 °C as shown in Figure 2. The values of the experimentally obtained mass losses are in good agreement with the theoretical mass losses (Δm_theoretic_ = 10.62%). The mass loss value for one mole of water is 3.54%. After removal of the hydrating water, anhydrous methotrexate presents good thermal stability in the range of 123–223 °C. The thermal decomposition of the active substance, the anhydrous form, takes place in the temperature range of 223–320 °C with a mass loss Δm = 40%. The decomposition residue is 45% of the initial mass. Heat flow (HF) curves of the methotrexate has four endothermic steps. The first two processes are accompanied by a loss of mass up to *T* = 132.5 °C, and at 196 °C a new endothermic peak appears, indicating the melting of the active substance in accordance with the values in the literature (195 °C). These results confirmed the purity of methotrexate as an active substance and demonstrated the melting of MTX, followed by decomposition [12].

#### 3.2.1. MTX in Solution

The injectable solution used is colored yellow because it contains methotrexate as the sodium salt of the active substance MTX (SA) analyzed above. This conclusion is also argued by the lack of the melting point of MTX (SA) and the presence on the Heat Flow curve of a weak endothermic process in the range 250–260 °C related to the melting of the sodium salt sample. The thermal analysis performed in the case of the recrystallized active substance from the solutions used for membrane synthesis revealed several decomposition steps, namely in the interval 46–174 °C a decomposition stage is observed that can be attributed to the loss of crystallization water. In this process, 8.3% of the sample mass is lost. Other decomposition steps take place over 313 °C by means of two clearly delimited processes on all the obtained thermoanalytic curves. The second process takes place in the interval 313–400 °C with a loss of 25% of the initial mass of the sample and then the third process is observed in the interval 400–500 °C with a loss of 34.6% of the mass of the sample. Endothermic processes accompany the last stages of decomposition. These processes will be taken as a benchmark in the thermal decomposition of binary mixtures with MTX as well as in synthesized membranes. All components were analyzed individually in order to highlight possible interactions between components initially in binary mixtures and then in membranes.

**Figure 3:**
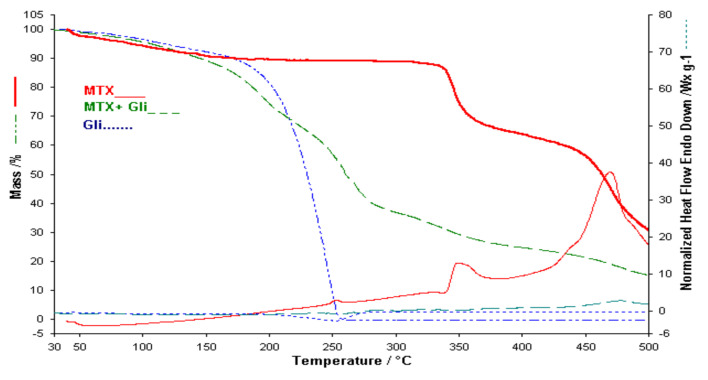
TGA/HF curves for MTX, Gli and Binary mixture.

Glycerol used in the synthesis of membranes for gelling purposes, presents in the thermogravimetric study two decomposition steps. The first is in the range of 34–156 °C with a mass loss of 8.25% that can be attributed to intramolecular water loss (Figure 3) [13]. The second endothermic process takes place in the interval 157–290 °C until the total loss of the sample mass. The process has a maximum at 253 °C and ΔH = 560 J g^−1^.

**Figure 4:**
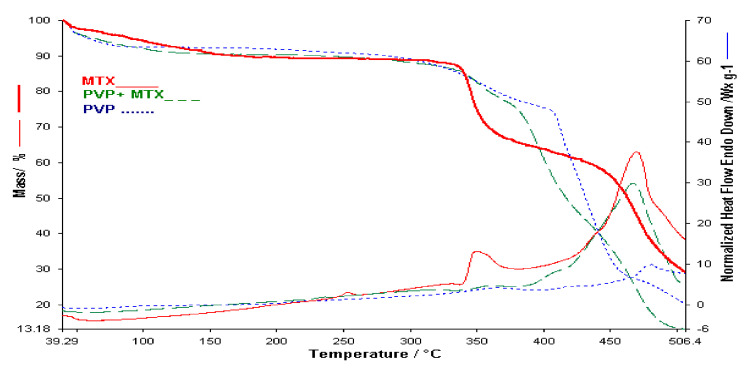
TGA/HF curves for MTX, PVP and Binary mixture.

In the case of PVP, the thermal degradation highlighted by thermal analysis, presents four decomposition processes. The first with a loss of 8.16%, the second in the range 271–406 °C with a loss of 15.9%, the third in the range 406–470 °C with a loss of 47.22% of the sample mass and the last process is not completed in the analyzed temperature range (Figure 4) [14,15].

**Figure 5:**
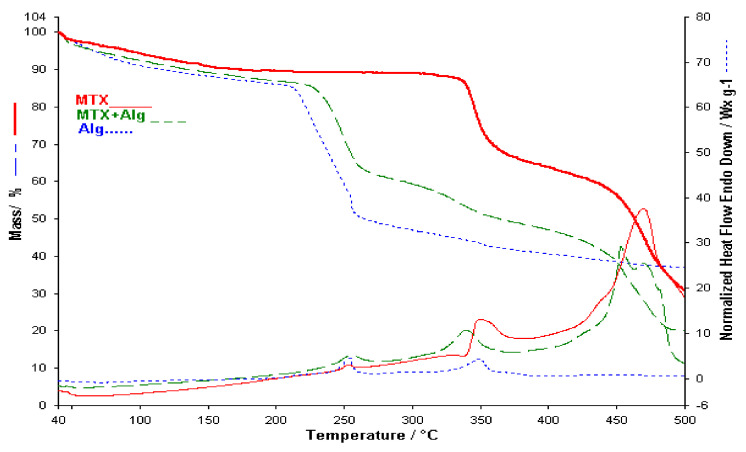
TGA/HF curves for MTX, Alg and Binary mixture.

As can be seen on the DTA (heat flow) curve of sodium alginate up to 500 °C, it has five distinct peaks (see Figure 5). An endothermic peak DTA at 85 °C is due to dehydration and due to this process at the TGA curve a mass loss of approximately 14.5% was observed, in the temperature range 35–210 °C. Major degradation of Na alginate occurred in the second temperature range, from 210 to 272 °C (weight loss of approximately 36.6%), with a maximum on the Heat Flow curve at 254 °C where volatile components are lost, chains are broken and sodium alginate is fragmented, stages visible on the TGA and Heat Flow (DTA) curves. This process is followed by two other processes with two exothermic peaks on the DTA curve with maxima at 297 and 349 °C [16,17]. The decomposition of Na alginate and its fragmentation into monomers occurred at temperatures above 400 °C. From Figure 5 it is clear that the processes, that took place in the temperature range from 210 to 500 °C, are exothermic. Alginate degradation was not completed within the studied temperature range (35–500 °C), observing a residue of 37% of the sample mass.

**Figure 6:**
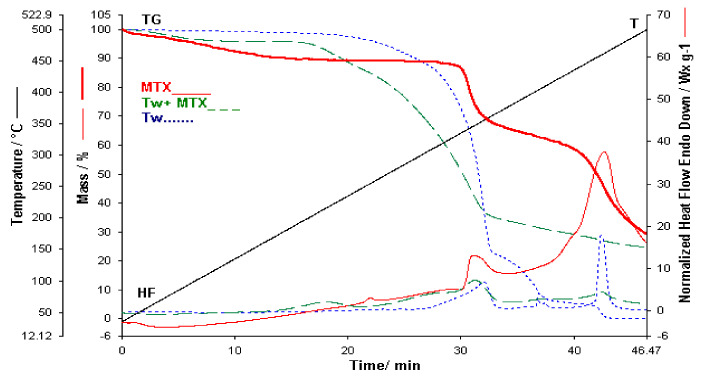
TGA/HF curves for MTX, Tw and Binary mixture.

Figure 6 shows the thermoanalytic curves obtained in the case of Tween which is used in the synthesis of membranes as a copolymer. The studied sample shows oxidative processes and mass loss in the oxidizing atmosphere used in the analysis. Three exothermic peaks are observed on the HF curve, the last being strongly exothermic, which coincides with the third stage of observable mass loss on the TG curve. The stages in the TG are, however, wide but can be separated based on the TG and DTG curves. The stages follow one another continuously, which indicate that no distinct intermediaries are formed. From DTG and TG it was found that the first exothermic reaction led to a weight loss of 66.5%, while the second exothermic stage led to an additional weight loss of 17.34% and the last of 4.7% from the sample mass. No residue is found at temperatures above 500 °C, which suggests a total oxidative decomposition of the sample. The first exothermic stage on the DTA curve in the temperature range between 150 °C and 260 °C corresponds to the initiation of the self-oxidation process, namely the introduction of oxygen as peroxide and the corresponding chain-reactions leading to primary oxidation products [18,19]. Oxidative rupture of the ethylene oxide chain is also known to be a potent exothermic process [20]. The second exothermic stage observed could be related to secondary degradation reactions such as recombination reactions. The presence of this strong exothermic process forced us to represent the curves as a function of time for a clearer visualization of the decomposition processes at high temperatures. Changes in the TGA curve begin only when volatile compounds are formed. This is in line with the complexity of a radical mechanism that first undergoes radical initiation, propagation, and termination, with the last two reactions leading to the formation of volatile compounds.

#### 3.2.2. The Study of Binary Mixtures

The study conditions were the same as in the case of the study on the individual components in order to facilitate the observation of possible changes. The thermal behavior of MTX in binary mixtures will be monitored, and in this way, the possible reactions or interactions between the substance used and the other components can be highlighted. The binary mixtures analyzed are composed of MTX and each component used in membrane synthesis. 

The first binary mixture studied by thermal analysis is the binary mixture made between MTX and Na Alginate in solid form where both the TGA curve and the HF curve show the decomposition stages of the individual components, which indicates that the two components do not present thermally induced interactions (Figure 5). The slight widening of the peak on the HF curve of the mixture can be argued by the combination of the thermal effects of both exotherms that accompany the individual decompositions of the two components. That is why the maximum is no longer the same but is shifted to temperatures lower by 20 °C. The thermal analysis of the binary mixtures was represented up to 500 °C in order to be able to better observe the last stages of decomposition of the components.

Binary mixture made between MTX and Glycerol (Figure 3) the endothermic peak present on the HF curve of Glycerol is combined with the exothermic peak present on the HF curve of MTX in the range 230–260 °C so that the processes of the individual components are not observed on the HF curve of the mixture. The other endothermic peaks present on the HF curve of MTX are poorly visible on the HF curve of the mixture, this can be argued due to a smaller amount of MTX present in the mixture. On the TG curve, the decomposition stage of the active substance is moved to lower temperatures attributed to a dispersion of the active substance. Regarding the influence of glycerol on the active substance in the membranes, it should not cause problems because glycerol is added in a very small amount in the membranes (in drops).

The HF curve of the MTX-PVP binary mixture contains the characteristic peaks of the individual components which argues that there are no interactions between the two components, therefore they can be used together within the membrane (see Figure 4). The HF curve of the mixture has a peak in the range 330–380 °C more blurred because the amount of active substance is much smaller than the analysis performed in the case of pure active substance [14].

In the case of the binary mixture conducted between MTX and *T*_w_ (Figure 6) [20], the thermal behavior of the individual components appears within the mixture, however in a smaller proportion due to the lower concentration of the components in the mixture up to 380 °C. Above this temperature the two exothermic peaks of the two components observed on the HF curves of both MTX and *T*_w_ are no longer visible on the mixture curve. It was expected that the decomposition of the mixture above 430 °C would show a powerful exothermic process. A different behavior of the mixture over 380 °C is also observed on the TGA curve, namely the mass loss stages related to process two and three are not visible in the case of copolymer *T*_w_, as well as the third decomposition process of MTX. Following the thermogravimetric study of the MTX-*T*_w_ mixture, it can be said that the two components of the mixture have interactions.

#### 3.2.3. Thermal Analysis of Membranes 

Alginate membrane obtained only from 1% Na alginate solution and glycerol (in drops) shows in the thermogravimetric study the same two stages of decomposition on the TGA curve as in the case of solid Na alginate but slightly modified on the DTG curve, namely one stage decomposition in the range of 25–101 °C with a mass loss of 9.4% due to water loss, and the second stage is a complex decomposition process consisting of several overlapping processes, as seen on the DTG and HF curve, one endothermic (in the range 101–206 °C) and the other weakly exothermic (in the range 206–250 °C). Obtaining the membrane improves the stability of the polymer chains so that the membrane is thermally stable in the range of 250–500 °C. The exothermic processes related to the loss of volatile components, the breaking of chains and the fragmentation of solid sodium alginate are no longer present on the HF curve. The last decomposition process in the range of 100–250 °C has a loss of 61% of the sample mass. The total mass loss of the alginate membrane, without active substance in the analyzed temperature range, is 75%.

**Figure 7:**
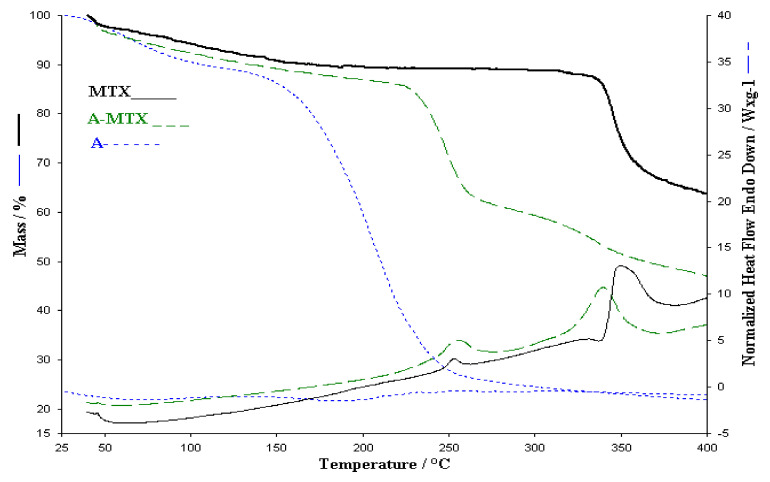
Comparative of TGA and HF for A, A-MTX and MTX.

Regarding the thermal analysis of the alginate membrane with MTX, on the TG curve it can be observed that it has a much higher stability compared to the alginate membrane without the active substance (Figure 7). The decomposition of the membrane with MTX starts at the same temperature as the alginate membrane but the main decomposition process starts above 240 °C. In addition, the decomposition process of the active substance above 330 °C is observed on the TGA curve of the membrane with MTX. On the HF curve of the membrane with MTX the same exothermic processes are observed as in the case of pure active substance but slightly displaced for the process that takes place around the temperature of 350 °C compared to membranes without active substance statement that can be argued by the dispersion of MTX within the membrane. Therefore, it can be said that the thermal analysis of the A-MTX membrane leads to the idea that the membrane has the active principle intact.

**Figure 8:**
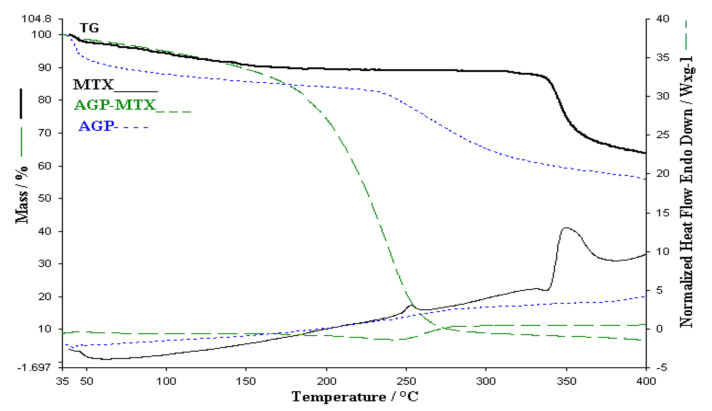
Comparative of TGA and HF for AGP membrane, AGP-MTX and MTX.

The thermal analysis performed in the case of the membrane obtained from alginates, PVP and MTX (Figure 8) highlights a decomposition that starts at 35 °C and ends at 280 °C with a continuous loss of mass. The thermal behavior of the membrane without active substance and of the active substance MTX is not observed. Decomposition processes cannot be separated. The exothermic processes of the active substance are not observed on the HF curve of the membrane with MTX. The thermal analysis performed in this case shows that there are interactions between membranes and MTX, although there are no interactions between MTX and the individual components in the membranes. Additional information will be provided from the FTIR study of the membranes and of the individual components.

**Figure 9:**
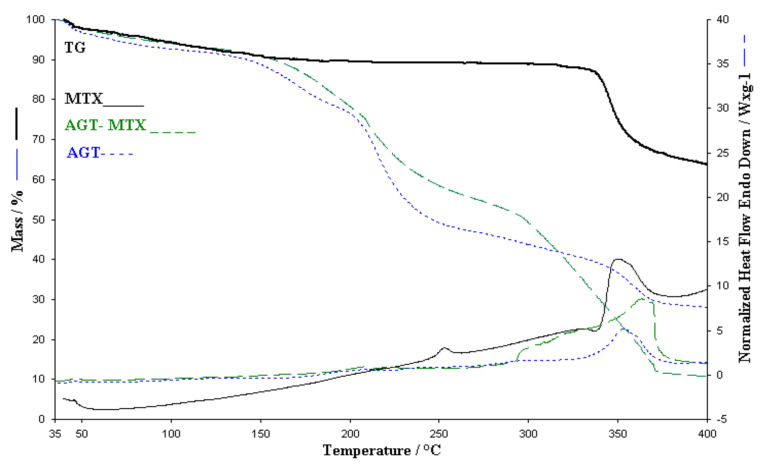
Comparative of TGA and HF for AGT membrane, AGT-MTX and MTX.

In the case of the membrane obtained from sodium alginates, glycerol and Tween (AGT), the thermal decomposition occurs via decomposition of a multiple stages (Figure 9) that does not end in the range of 25–500 °C. The total mass loss is 76% of the sample mass. In the range of 50–116 °C there is a loss of water (4.18%), in the interval 118–190 °C there is a decomposition process with a loss of 13.24% of the sample mass followed by a more important mass loss of 31.7% in the range of 190–72 °C with a maximum at 215 °C. There are also three stages of decomposition with losses of 5.5% and 12.62%, this being accompanied by a strong exothermic process with a maximum at 354 °C. The rest of the decomposition stages are accompanied by endothermic processes. The stages of decomposition are difficult to separate which leads us to the idea of complex processes.

Thermal analysis of the ATG-MTX membrane revealed three decomposition stages leading to a loss of 90% of the total mass in the studied range. Comparing the HF and TGA curves shown in Figure 9 it can be said that the thermal analysis performed in the case of ATG-MTX membrane contains the decomposition processes of the membrane and the active substance, the exothermic process of the active substance around 350 °C is also present in the membrane, is more extensive containing the process of membrane decomposition which leads us to the idea that the membrane contains the active principle intact [20]. Although in the study of the binary mixture between *T*_w_ and MTX we noticed that MTX interacts with *T*_w_, in the case of alginate-based membrane and *T*_w_ the conclusion is different, namely the addition of *T*_w_ in the membrane blocks the interaction between MTX and *T*_w_ because a copolymer is obtained between alginate and *T*_w_ that does not interact with the active substance [21,22].

### 3.3. FTIR Study

**Figure 10:**
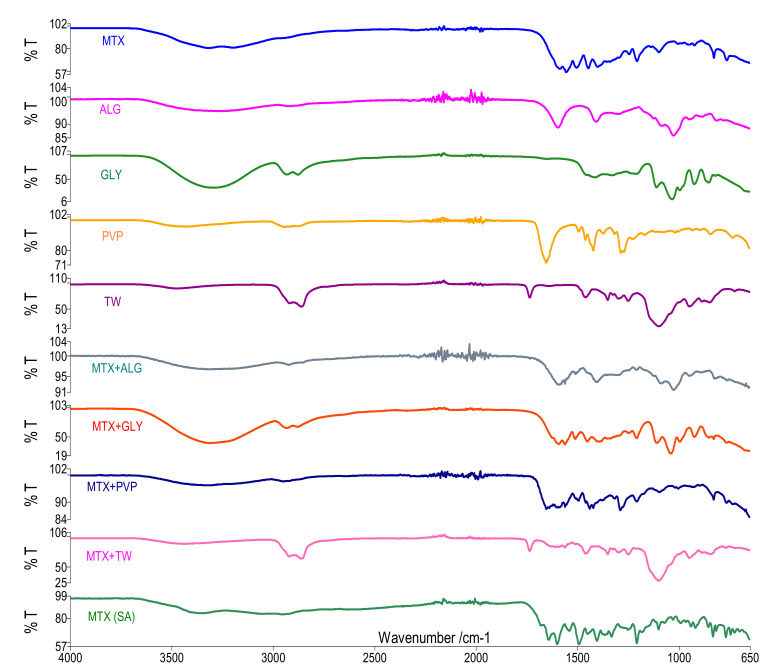
FT-IR spectra.

In the FTIR study, the FTIR spectra of the individual components were compared in Figure 10, following closely the presence of the main vibrations within the active substance.

The UATR-FTIR spectrum of pure methotrexate-active substance MTX(AS) shows characteristic absorptions band as a broad signal at 3350 cm^−1^ (O–H stretching from carboxyl groups superposed with the O–H stretching from crystallization water), at 3080 cm^−1^ (primary amine N–H stretching), at 2950 (C–H vibration from CH_3_ group), at 1670–1600 cm^−1^ assigned to C=O stretching (–C=O stretching from carboxylic group and C=O stretching from amidic group, so the C=O band is splinted into a doublet in the MTX(AS) sample). The bands corresponding to N-H bending from amidic group appear in the 1550–1500 cm^−1^ spectral range, partly overlapping with the aromatic –C=C stretching. Another prominent bands, such as 1400–1200 cm^−1^ correspond to –C–O stretching from carboxylic group, 940 cm^−1^ to O–H bending out of plane and 830 cm^−1^ to C–H - 2-adjacent hydrogens on an aromatic ring, para substitution [12]. 

The characteristic bands of MTX appear very well represented in the case of the MTX spectrum obtained from the recrystallization of the solution used for membrane synthesis. The MTX spectrum obtained from the solution used for membrane synthesis shows stretching vibrations characteristic of the weak C–H bond at about 2900 cm^−1^, the bands characteristic of the OH group at 3200 cm^−1^ are also observed, the C=C bonds in the aromatic rings are presented at 1445 cm^−1^ as well as the vibration related to the C–N connection at 1100 cm^−1^. It can be stated that the vibrations associated with carboxylic acid and carboxylate have two characteristic marks at 1250 and 1400 cm^−1^ corresponding to the stretching bands C=O and C–O. On the other hand, the displacement of the O–H bond disappears [23]. All the bands identified in the FTIR spectrum are in good agreement with the molecular structure of MTX and confirm its purity.

#### 3.3.1. FTIR Study for Binary Mixtures

To ease the interpretation of the results obtained from the FTIR study, the spectra of the individual components are represented in comparison with the FTIR spectrum of the binary mixture (Figure 10). The spectrum of solid sodium alginate shows the absorption bands of the hydroxyl, ether and carboxyl functional groups. Vibration of the O–H bonds occurred in the range of 3000–3600 cm^−1^. Vibrations related to aliphatic C–H bonds were observed at 2920–2850 cm^−1^. Intense bands observed at 1600 and 1406 cm^−1^ were attributed to asymmetric and symmetrical stretching vibrations in the carboxylates ion. The bands between 1100 and 950 cm^−1^ were attributed to the C–O stretching vibration of C–O as well as to the deformation of the C–C–H and C–O–H bonds [23]. Regarding the spectrum of the binary mixture, the presence of the characteristic peaks of MTX is observed, namely the vibrations characteristic vibrations of the C=O, C–N bonds as well as of the C–H bonds at the carboxylic group, N–H amine. Some MTX bands are covered by the characteristic bands of alginate, considering the presence of the same types of bonds (see carboxylate group) [24].

The FTIR spectrum of glycerol shows peaks of several functional groups. The O–H stretch frequency was observed at 3300 cm^−1^ while the C–H stretch was clearly highlighted in the region 2880–2950 cm^−1^. The bending of the C–O–H group was also observed in the region from 1400 to 1450 cm^−1^, the displacement of the C–O bond of the primary alcohol is represented by the intense band from 1030 cm^−1^ (Figure 10). In the case of the MTX + Gly mixture, the spectrum highlights the characteristic bands of MTX very well represented in the area 1550–1100 cm^−1^. The spectral area of the mixture covered by the FTIR spectrum of Gly is 1100–900 cm^−1^. Following the spectral study of the MTX + Gly mixture, it can be said that Gly can be used in membranes. However, we can also consider the results of the thermogravimetric study where interactions between components were observed. These interactions may exist at the C–N and C–O bonds that cannot be attributed to the mixture. It is recommended to use a small amount so as not to influence too much the concentration of the active substance in the membranes.

In the case of the MTX + PVP mixture, the bands characteristic of the active substance is visible next to the band from 1660 cm^−1^ attributed to the stretching vibration of C=O, as well as the peak at 2950 cm^−1^ attributed to the asymmetric stretching of the CH_2_ vibration in the PVP skeleton chain. From the point of view of the FTIR study, we can say that there are no interactions between MTX and PVP in the binary mixture (Figure 10) [14,21,22].

Tween’s FTIR spectrum highlights hydrogen-bound OH stretching to 3482 cm^−1^, 2863 and 2921 cm^−1^, which can be attributed to asymmetric and symmetrical methylene stretching vibrations at the specific carbonyl group of 1734 cm^−1^ from R–CO–OR, at 1640 a wide band corresponding to the extent of the carbonyl and at 1097 cm^−1^ the extended vibration of –CH_2_–O–CH_2_– (Figure 10) [23]. As for the spectrum of the mixture, it contains the Tw bands mentioned above, but also the characteristic MTX bands, however at slightly displaced wavelengths and weaker in intensity. Thus, the band specific to the NH amine bond appears at a higher wave number and with a very low intensity, the band from 1400 cm^−1^ is not observed, but the band from 1450 cm^−1^ is observed at a higher wave number. The vibration at 1240 cm^−1^ is present claiming the presence of the unchanged C=O bond, the band at 940 cm^−1^ related to the displacement of OH in MTX is no longer present in the mixture, but a band related to both components is present at 945 cm^−1^. At the same time, the vibration of the OH bond in MTX and *T*_w_ is also present in the mixture, but much weaker, which leads us to the idea of a reaction at the level of this bond.

In the case of the FTIR study of binary mixtures made between MTX and the individual components used for membrane synthesis, the presence of characteristic MTX bands at 3200 cm^−1^ related to OH vibration is very well represented in the case of mixtures between MTX and Alginate, Glycerol, PVP being much less pronounced in the case of the mixture between MTX and *T*_w_. The C=C bonds in the aromatic rings are present at 1440–1490 cm^−1^, and the vibrations related to the C–N bond at 1100 cm^−1^ are very visible in the same mixtures. The vibrations from 1400 cm^−1^ corresponding to the stretching bands C–O in all mixtures are very well represented. The FTIR spectrum of the MTX-*T*_w_ mixture shows bands at different wave numbers, which argues the results of the thermogravimetric study, respectively an interaction between the two components, interactions that are not observed in the case of the other binary mixtures studied [24,25,26].

#### 3.3.2. FTIR Study of Membranes

A comparative study [27] of the membrane obtained from alginate, glycerol, MTX with and without active substance in membrane, it can be seen that the membrane with MTX contains the characteristic peaks of the active substance, namely the characteristic vibration of the OH bond in MTX is present along with that of the Alginate. The characteristic bands of the active substance from 1450, 1500, 1200, 1100, 1000, 920, and 825 cm^−1^ are also present. The presence of these bands argues for the existence of C=O, C–N, and C–O within the active substance.

In the case of the FTIR spectrum [27] of the AGP-MTX membrane (Figure 10), the characteristic band MTX is observed in the area 3300 cm^−1^, as well as the characteristic bands of the C-H bonds in the AGP membrane in the range 2880–2950 cm^−1^. At the same time, the characteristic bands of the active substance at 1250, 1100, 1000, 920, and 825 cm^−1^ are well highlighted. The peaks in the area 1580–1400 cm^−1^ are less visible in the membrane spectrum. FTIR study of the AGP-MTX membrane may lead to the idea of an interaction between the membrane and the active substance via carboxylate group.

The comparative FTIR spectrum of the active substance and of the AGT-MTX (Figure 10) membrane shows the characteristic peaks of MTX, respectively, at 3200 cm^−1^ related to the OH vibration, but covered by the water present in the membranes. The C=C bonds in the aromatic rings are present at 1440–1490 cm^−1^ as well as the vibration related to the C–N bond at 1100 cm^−1^, which is very clearly visible in this membrane together with the vibration related to the carboxylate group from 1250 to 1400 cm^−1^.

### 3.4. SEM Analysis

**Figure 11:**
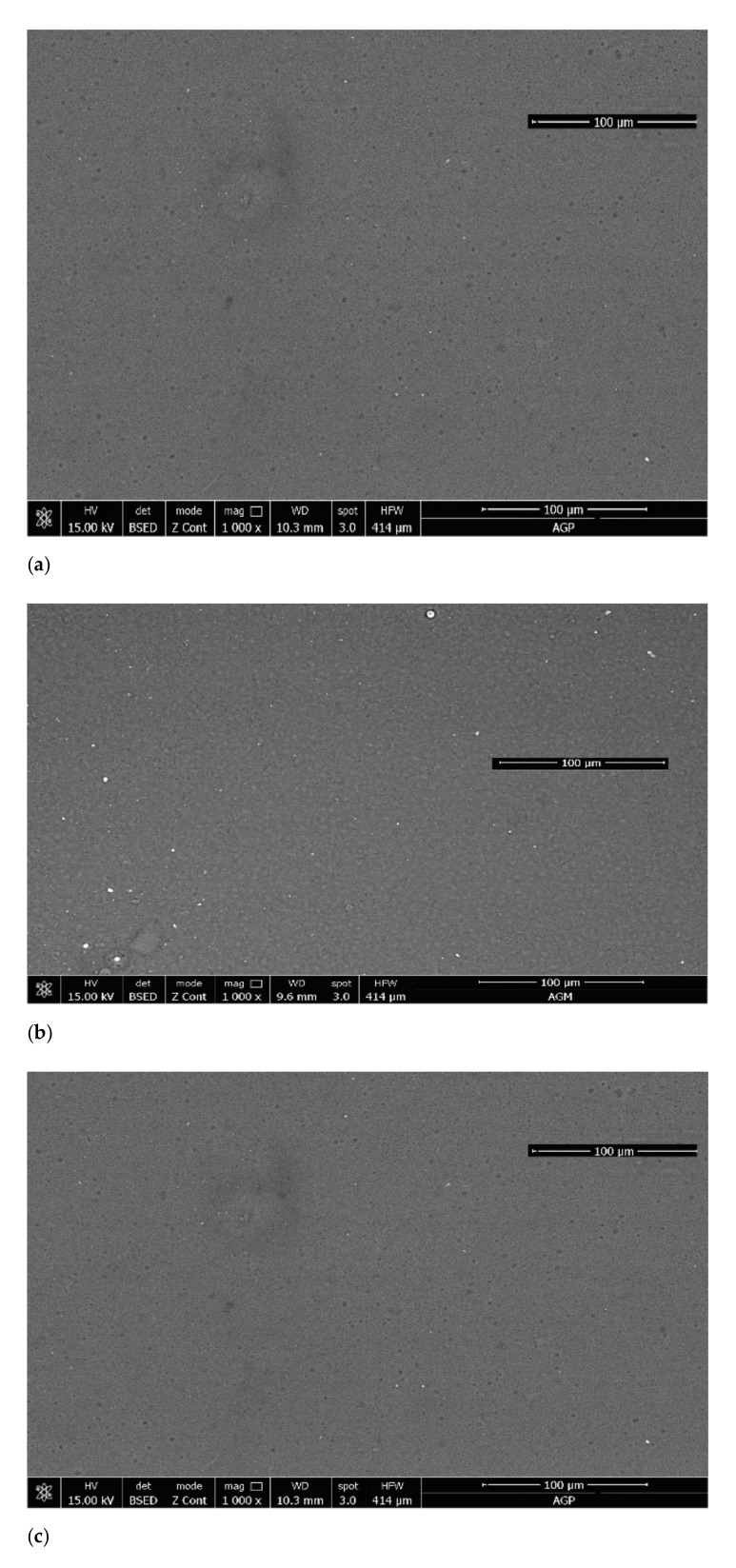

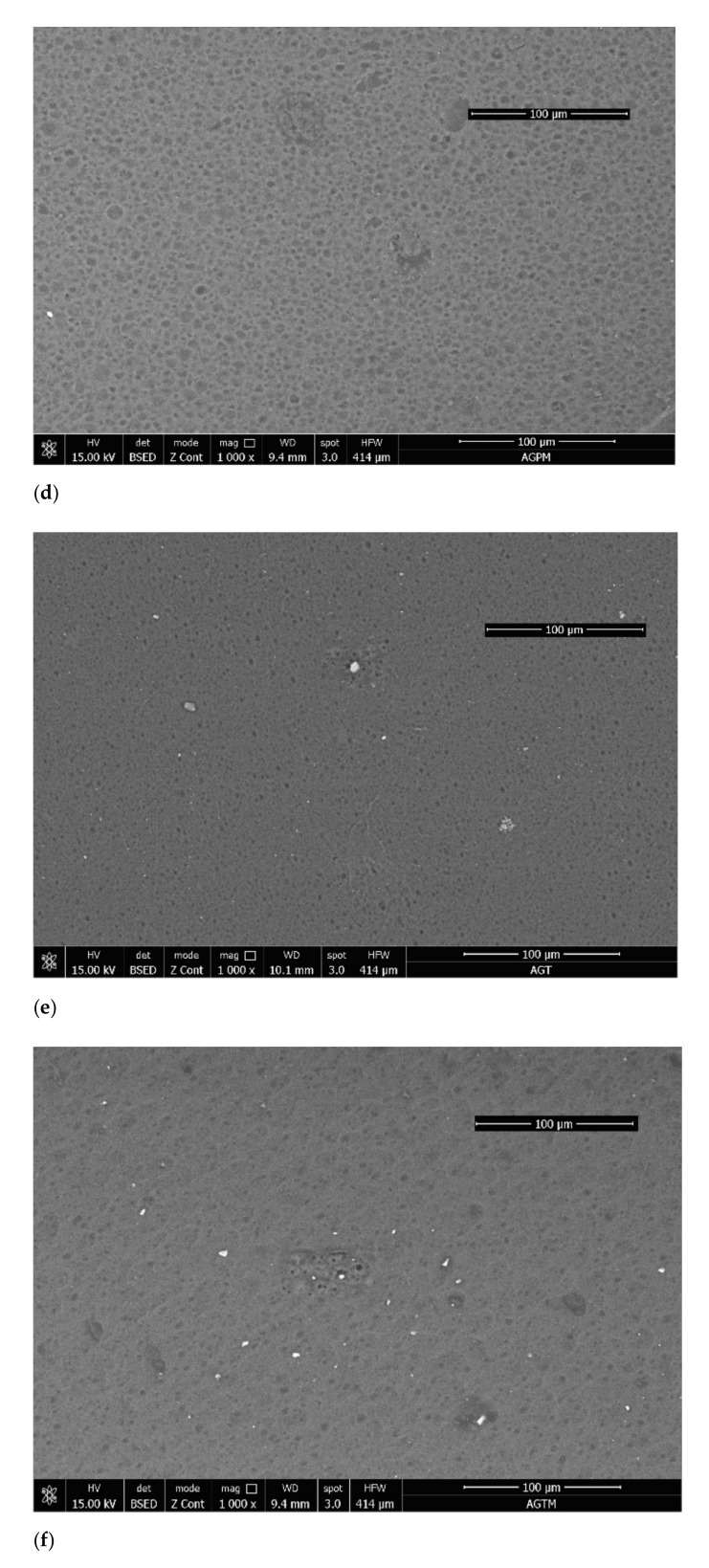
SEM image of the (**a**) AG, (**b**) A-MTX, (**c**) AGP, (**d**) AGP-MTX, (**e**) AGT and (**f**) AGT-MTX membrane.

Following the SEM analysis (Figure 11), comparative images of membranes without active substance and those with active substance are presented. The analysis highlights that the AG, AGP and AGT membrane are homogeneous with a lower porosity in the case of the AG membrane and with a higher porosity in the case of the other membranes. At the first observation it can be said that the membranes without active substance have a homogeneity that varies as follows: A < AGP < AGT. Regarding the membranes with active substance, it can be observed that all membranes present an image with a homogeneous distribution of the active substance [28,29]. 

### 3.5. UV/Vis Analysis

An MTX stock solution at 10 mg mL^−1^ was produced in a Sodium Chloride solution. All absorbance measurements were taken in a 1 cm of path-length cuvette at room temperature, at wavelengths between 190 and 400 nm using Sodium chloride solution 0.9% as a blank. 

For the analysis of membranes with MTX, an amount of each type of membrane was weighed and then solubilized in 0.9% NaCl solution. The active principle inside the membranes was highlighted. The UV spectra of the active substance, of the membranes without active principle were obtained and compared with the UV spectra of the membranes with MTX (Figures S1–S7). In the case of all membranes that incorporated MTX, the presence of MTX at the wavelength of 270 nm was highlighted.

## 4. Conclusions

The study presents the synthesis and characterization of several types of membranes based on Alginate in order to choose the best membrane that can be used for the transdermal release of MTX. During the synthesis step, alginate was used as a biopolymer material to which other polymers such as PVP, PVA, and Tween and Carbopol were added. Following the synthesis, the membranes that meet the conditions of homogeneity and stability were chosen. In order to argue for the lack of interactions between the basic components within the membranes and the base substance, studies were performed by TGA/DTG/HF (DTA) and FTIR methods on binary mixtures (1:1) following which it was observed that the active substance has interactions with glycerol (which is added for gelling purposes) and with Tween. From this part, it was deduced that glycerol can be used in syntheses because a very small amount is needed that would not influence the concentration of MTX available in the membrane. The studies were then extended to the membranes obtained and compared with the membranes without active substance. The analyses were completed with the results of the UV-Vis and SEM study. Following these studies, it was concluded that membranes obtained from Alginate/Alginate and Tw can be used for the release of methotrexate. Regarding the AGP-MTX membrane, it presents interactions with the active substance, carboxylate group interactions argued by TGA and FTIR studies, interactions that occur in aqueous medium. The SEM analysis revealed the achievement in all cases of homogeneous membranes. Regarding the UV-Vis analysis, it can be said that, when the membranes dissolve, the active substance is released so that it could be highlighted. In conclusion, the A-MTX and AGP-MTX membranes can be used for the transdermal release of methotrexate.

## Figures and Tables

**Table 1:** The composition, notation of membranes and their appearance.

Composition.	Notation of Membranes	Membrane Appearance.	Composition	Notation of Membranes	Membrane Appearance.
Sodium alginate, Glycerol	A	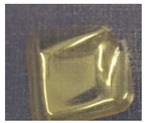	Sodium alginate, Glycerol, PVP	AGP	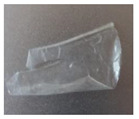
Sodium alginate, Glycerol + MTX	A-MTX	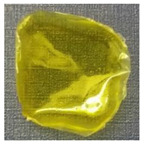	Sodium alginate, Glycerol, PVP + MTX	AGP-MTX	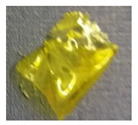
Sodium alginate, Glycerol, Tween 20	AGT	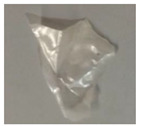	Sodium alginate, Glycerol, Carbopol	AGC	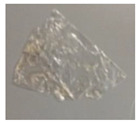
Sodium alginate, Glycerol, Tween 20 + MTX	AGT-MTX	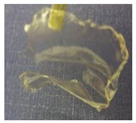	Sodium alginate, Glycerol, Carbopol+ MTX	AGC-MTX	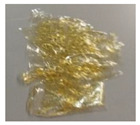

## Data Availability

The data presented in this study are available on request from the corresponding author.

## Supplementary Materials

The following are available online at https://www.mdpi.com/2073-4360/13/1/161/s1, Figure S1: UV-Vis spectrum of AG, Figure S2: UV-Vis spectrum of AGM, Figure S3: UV-Vis spectrum of AGP, Figure S4: UV-Vis spectrum of AGPM, Figure S5: UV-Vis spectrum of AGT, Figure S6: UV-Vis spectrum of AGTM, Figure S7: UV-Vis spectrum of MTX.
