# Supplementary material for: Formulation and Characterization of Alginate-Based Membranes for the Potential Transdermal Delivery of Methotrexate
Source: Polymers (Basel). 2021 Jan 4;13(1):161. doi: 10.3390/polym13010161 (PMC7794806; doi:10.3390/polym13010161)
Supplement: Supplementary file 1 [file polymers-13-00161-s001.pdf]

polymers

MDPI

Supplementary Materials

Formulation and Characterization of Alginate-Based Membranes for the Potential Transdermal Delivery of Methotrexate

Dorothea Bajas [1,†], Gabriela Vlase [1,†], Mădălina Mateescu [1], Oana Alexandra Grad [2], Mădălin Bunoiu [3,*], Titus Vlase [1,*] and Claudiu Avram [4]

[1] Research Centre for Thermal Analysis in Environmental Problems, West University of Timisoara, Pestalozzi Street 16, 300115 Timişoara, Romania; dorothea.bajas@gmail.com (D.B.); gabriela.vlase@e-uvt.ro (G.V.); madalina.mateescu@e-uvt.ro (M.M.); titus.vlase@e-uvt.ro (T.V.)
[2] Research Institute for Renewable Energy, Politehnica University of Timişoara, Piata Victoriei, No. 2, 300006 Timisoara, Romania; oana.grad@upt.ro
[3] Faculty of Physics, West University of Timisoara, B-dul V. Parvan No.4, 300223 Timisoara, Romania; madalin.bunoiu@e-uvt.ro
[4] Physical Therapy and special motricity department, West University of Timisoara, B-dul V. Parvan No.4, 300223 Timisoara, Romania; claudiu.avram@e-uvt.ro
* Correspondence: madalin.bunoiu@e-uvt.ro (M.B.); titus.vlase@e-uvt.ro (T.V.); Tel.: 0040256592166 (M.B.); 0040256592627 (T.V.)
† Equal contributions first author.

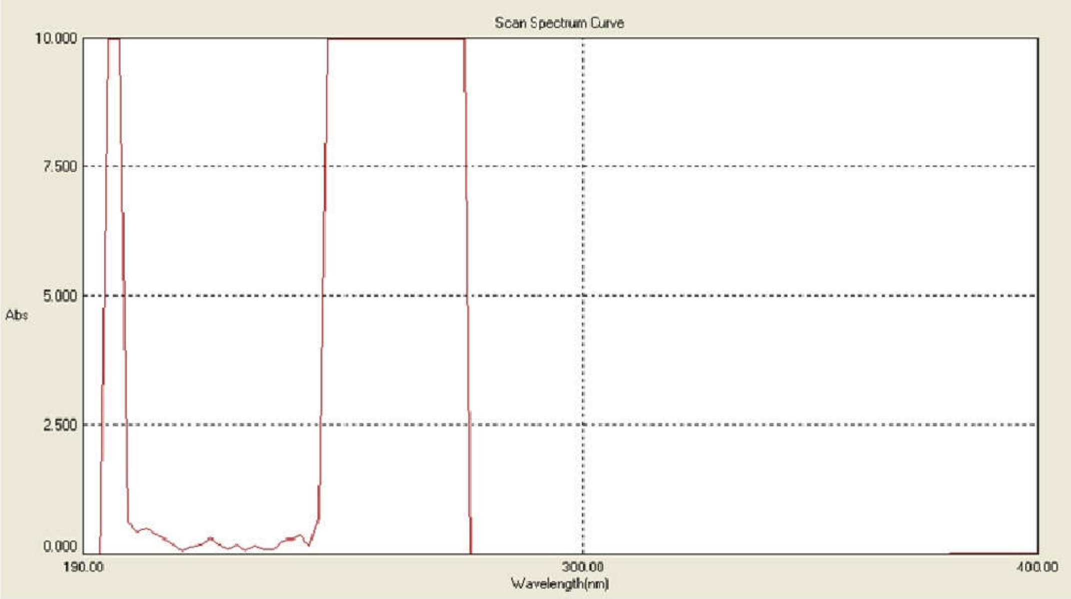

Figure S1. UV-Vis spectrum of AG.

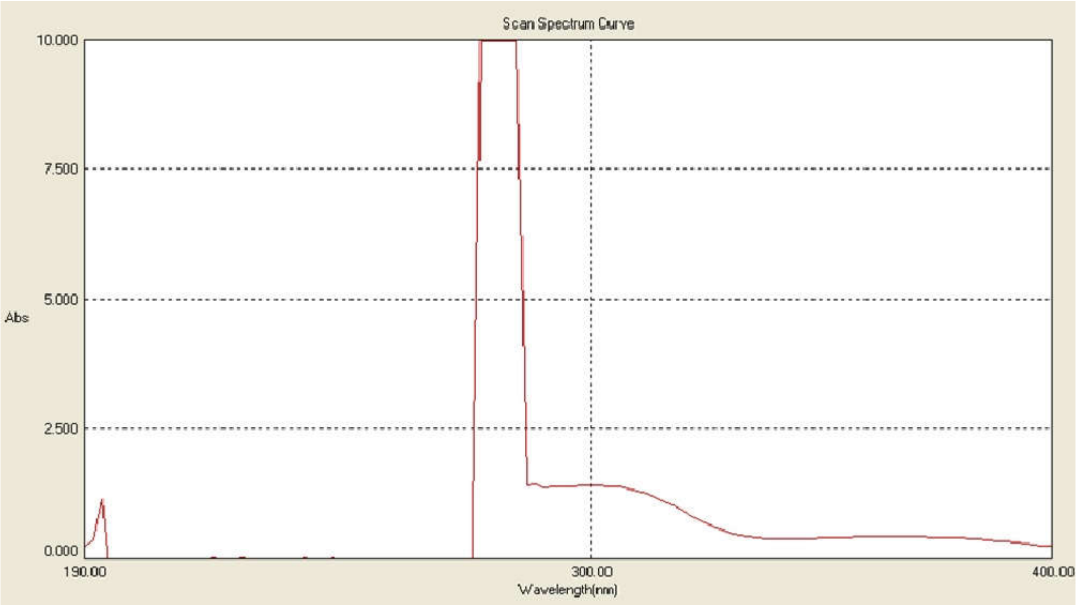

Figure S2. UV-Vis spectrum of AGM.

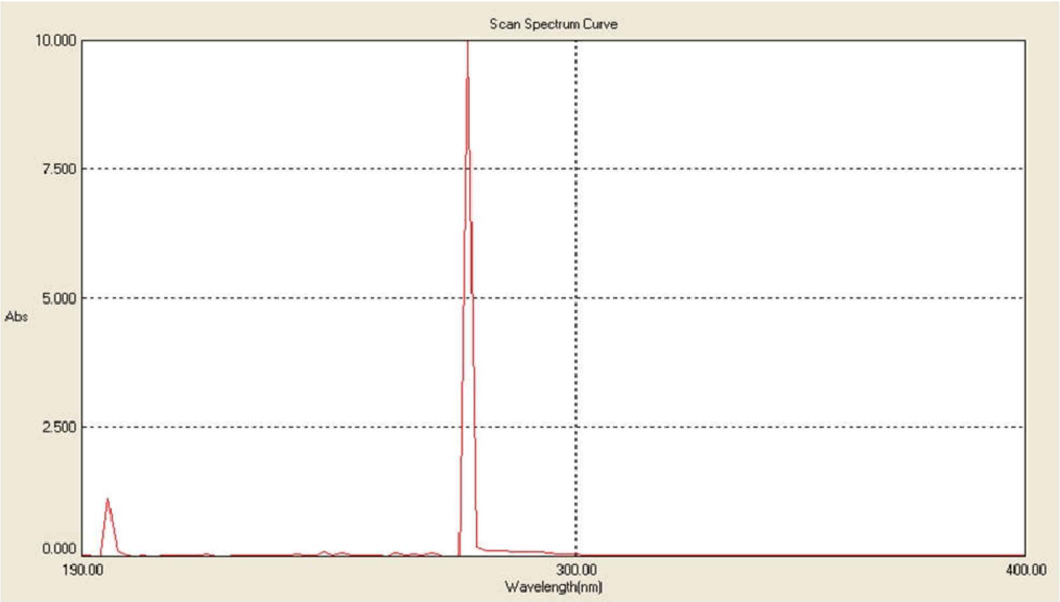

Figure S3. UV-Vis spectrum of AGP.

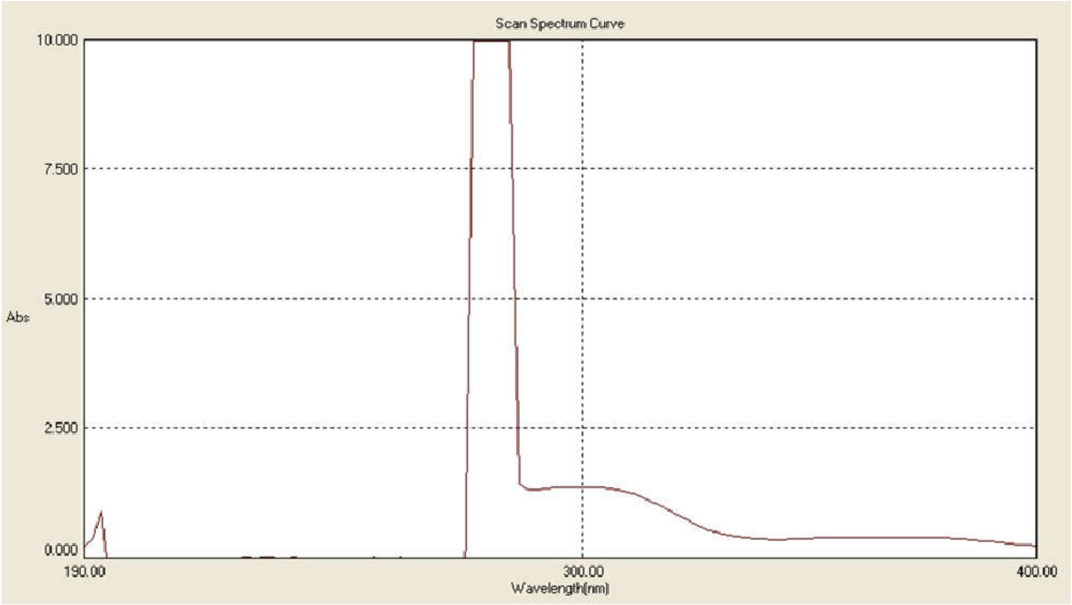

Figure S4. UV-Vis spectrum of AGPM.

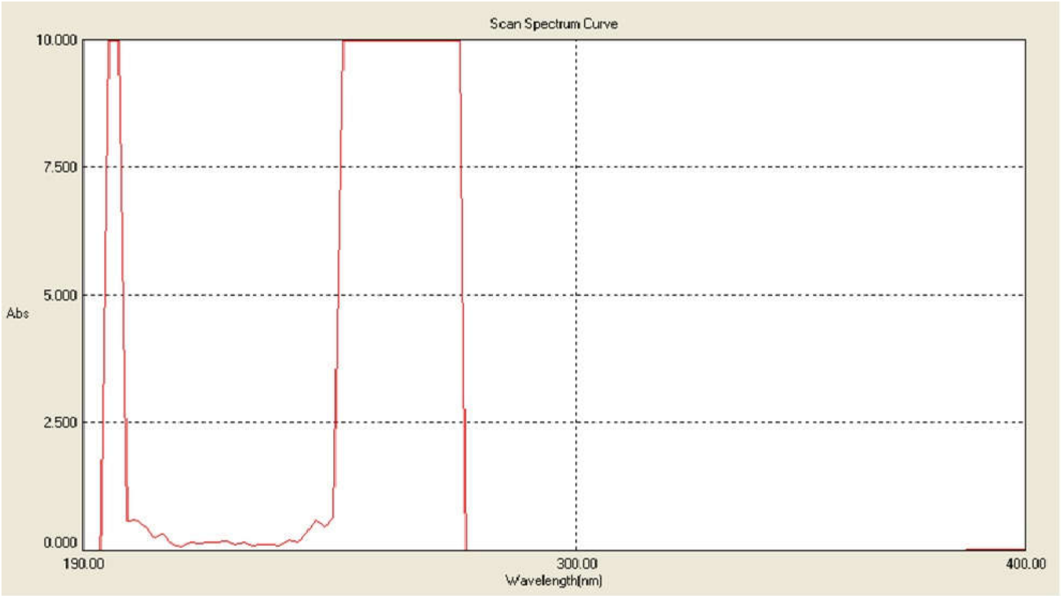

Figure S5. UV-Vis spectrum of AGT.

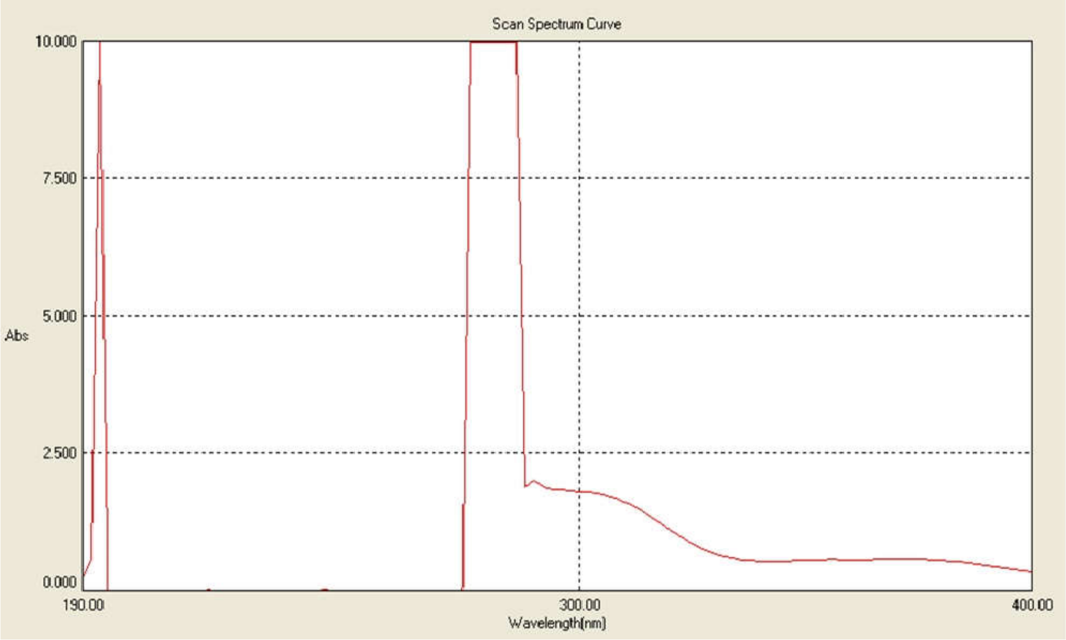

Figure S6. UV-Vis spectrum of AGTM.

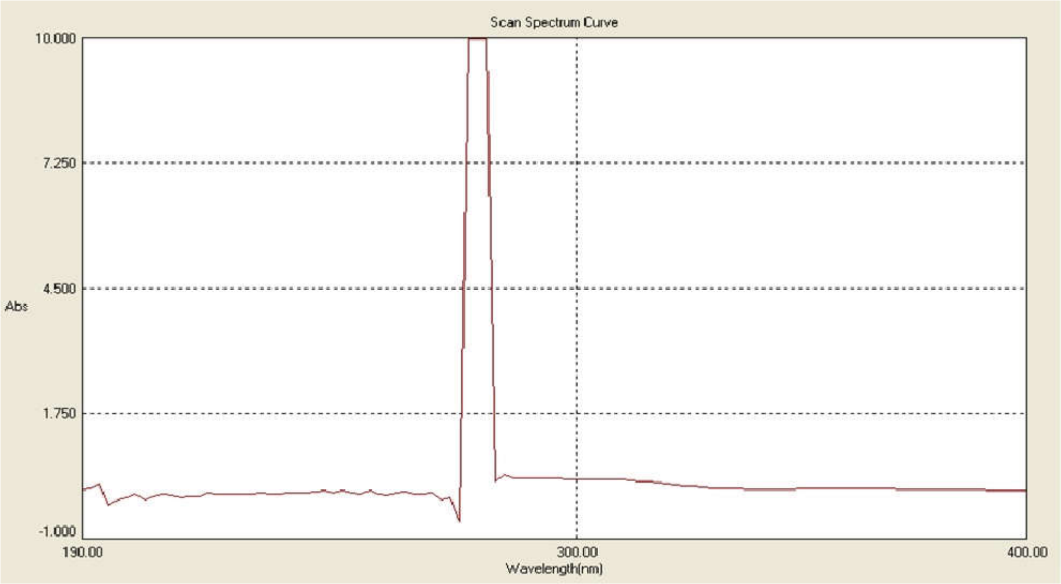

Figure S7. UV-Vis spectrum of MTX.
